# Hepatocellular Carcinoma: First Manifestation as Solitary Humeral Bone Metastasis

**DOI:** 10.1155/2020/8254236

**Published:** 2020-12-01

**Authors:** Sumera Bukhari, Kristine Ward, Michael Styler

**Affiliations:** ^1^Cambridge Health Alliance-Harvard Medical School, Boston, Massachusetts, USA; ^2^Pennsylvania Hospital-Penn Medicine, Philadelphia, Pennsylvania, USA; ^3^Fox Chase-Temple University Hospital, Philadelphia, Pennsylvania, USA

## Abstract

Hepatocellular carcinoma (HCC) most commonly presents with abdominal pain or mass, fever of unknown etiology, weight loss, and decompensation of known liver disease or at an asymptomatic stage through surveillance. Rarely, presenting symptoms can be exclusively related to extrahepatic metastases. Herein, we write a case of a patient with no known liver disease, presenting with a pathological fracture of the proximal humerus bone secondary to a massive solitary metastasis from HCC. This case represents an unusual appendicular skeletal metastasis in a patient with unknown primary HCC, successfully treated with sorafenib. The prognosis of HCC patients with extrahepatic metastasis is poor, and in the presence of bone metastases, the mean survival rate is severely reduced. However, the multikinase inhibitor sorafenib has been the standard of treatment. Recently, there has been developments of other therapeutic class of drugs (i.e., immune check inhibitors), which have shown promising benefits and better side effect profiles. Still, there is a need for further studies, owing to challenges in recognizing cellular and molecular markers.

## 1. Introduction

Hepatocellular carcinoma (HCC) is the most common primary liver malignancy. It is the third and seventh most prevalent cancer worldwide in men and women, respectively, and is the fourth most leading cause of cancer-related death in the world [1, 2]. Major risk factors include hepatitis B, hepatitis C, alcoholic liver disease, and nonalcoholic steatohepatitis. The distribution of these risk factors among patients with hepatocellular carcinoma is significantly different, depending on geographic region and race or ethnicity [3]. Most commonly, patients with HCC present with abdominal pain or mass, fever of unknown etiology, and weight loss or decompensation of known liver disease, while up to one-fourth are diagnosed at an asymptomatic stage through surveillance [4]. Rarely, patients may have initial symptoms exclusively related to extrahepatic metastases [5]. At the time of HCC diagnosis, only 5 to 15% of cases have the extrahepatic spread [6]. The most frequent sites of extrahepatic metastasis are the lung, lymph node, adrenal gland, and bone [7]. HCC metastasis to the bone occurs less frequently than other cancers and is considered a rare primary form of presentation [8]. Most reported bone metastasis cases are accompanied by either multiple metastatic spreads elsewhere in the body or previously known HCC. However, in our patient, the bone metastasis was isolated to humerus bone and was the first presentation of HCC. To the best of our knowledge, there are few published cases in the literature to date with unusual bone metastasis to the distant appendicular skeleton presenting as the first manifestation of an unknown primary HCC [9–13]. Herein, we write a case of a patient with no known liver disease presenting with a pathological fracture of the upper arm bone, who was found to have a massive solitary metastasis of HCC to the proximal humerus bone.

## 2. Case Presentation

**Figure 1:**
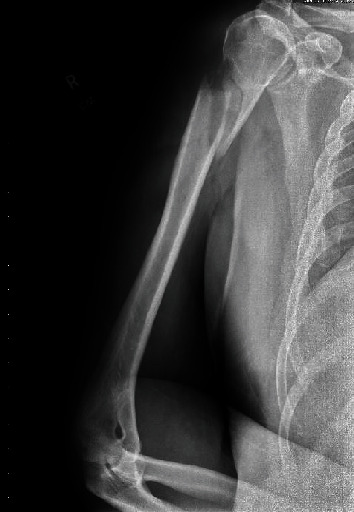
X-ray of the shoulder showing spiral fracture of proximal diaphysis of the humerus with displacement and angulation of the distal fractured bone.

A 59-year-old male with a significant history of chronic alcoholism presented to the emergency room after an upper arm fracture on light gardening. X-ray of the shoulder showed a spiral fracture of the proximal diaphysis of the humerus with displacement and angulation of the distal fractured bone (Figure 1). During the orthopedic surgery evaluation, the patient reported dyspnea on exertion and increasing abdominal girth. These alarming symptoms prompted a referral to cardiology for preoperative clearance. The patient got admitted for the pre-op workup. His past medical history was significant only for chronic alcohol abuse. He denied smoking and use of illicit drugs. His family history was noncontributory and negative for bone or liver disease. His vitals on admission were the following: temperature of 97.1, blood pressure of 160/65 mmHg, respiratory rate of 18 breaths per minute, and pulse rate of 60 beats per minute. Pertinent physical findings included mildly enlarged liver on palpation, grade IV/VI systolic murmur at the aortic area, and scattered basilar rales. There was no splenomegaly, jaundice, or lymphadenopathy.

**Figure 2:**
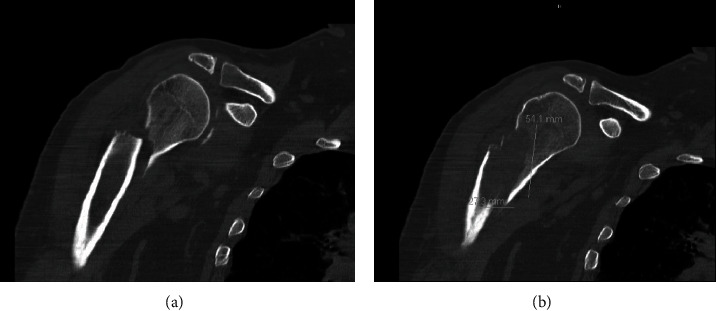
(a) Computerized tomography scan of shoulder showing an oblique comminuted fracture of the proximal diaphysis of humerus. (b) An ovoid lucency measuring approximately 5.5 × 2.8 cm within the proximal humerus shaft at the site of fracture.

**Figure 3:**
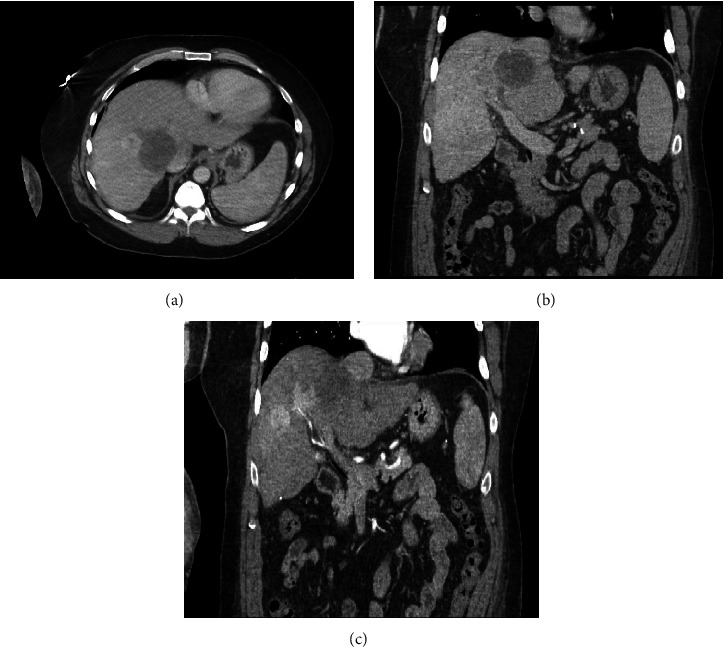
(a–c) Computerized tomography scan of abdomen and pelvis showing four liver lesions typical of HCC, with one hypoattenuating large lesion atypical for HCC.

Laboratory workup showed hemoglobin 11.8 g/dL, white blood count 5,700/mm^3^, platelets 115,000/mm^3^, BUN 14 m/dL, creatinine 0.62 mg/dL, calcium 8.7 mg/dL, albumin 2.7 g/dL, total proteins 8.4 g/dL, prothrombin time 14 seconds (control 10-13 seconds), activated partial thromboplastin time 34 sec (control 27-37 seconds), alkaline phosphatase 172 IU/L, ALT 71 IU/L, AST 107 IU/L, LDH 224 IU/L, total bilirubin 1 mg/dL, alpha-fetoprotein 8.7 ng/mL, hepatitis C antibody reactive, and hepatitis C viral load of 1,626,714. Computerized tomography (CT) scan of the shoulder (Figure 2(a)) showed an oblique comminuted fracture of the proximal humerus shaft as reported on X-ray in the emergency room. However, the fracture appeared to traverse through an ovoid lucency measuring approximately 5.5 × 2.8 cm within the proximal humerus shaft (Figure 2(b)). No discrete tumor or soft tissue mass was seen but was likely obscured by hemorrhage from the fracture. CT scan of the abdomen and pelvis (Figures 3(a)–3(c)) revealed cirrhosis with portal hypertension, showing four liver lesions, with the largest measuring 3.1 cm, typical of HCC and another one hypoattenuating large lesion, measuring 5.8 × 5.2 cm, atypical for HCC. Staging scans did not reveal metastatic disease in the chest.

**Figure 4:**
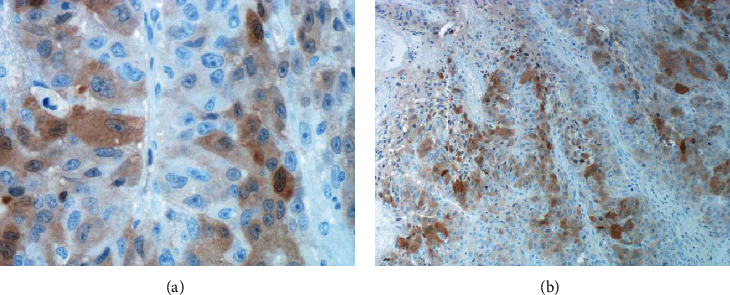
(a–b) Biopsy results showing the presence of HCC (high-power arginase stain).

The differentials considered based on radiological features were a primary bone tumor or metastasis of an unknown primary or hepatocellular carcinoma. The patient underwent an open reduction and internal fixation of the right humerus with a bone biopsy at the fracture site with no postoperative complications. The biopsy results showed the presence of HCC (Figures 4(a) and 4(b)). The patient was recommended to start radiation to the humerus and sorafenib after recovery from surgery.

## 3. Discussion

The skeleton is the third most common metastasis location after the lung and liver [14, 15]. Skeletal metastasis of unknown primary (SMUP) is a mysterious, unusual metastatic tumor entity without any known anatomic primary sites. Skeletal metastasis of unknown primary (SMUP) imposes a challenge in the clinical management of patients diagnosed with bone metastases. The approach to the management of these patients has changed considerably in the last few years. However, SMUP patients need more comprehensive and tailored treatment. Incorporating genetic and molecular features in a multistage diagnostic workup is essential for characterizing the biological SMUP profile to direct therapeutic decisions. SMUP is a comprehensive diagnosis and treatment plan that must be adapted to different pathophysiological complexities [16].

Metastasis from HCC tends to travel to the axial skeleton. The most common bone metastasis sites are the vertebra, followed by the pelvis, rib, and skull [8]. Metastatic involvement is rarely found in the appendicular skeleton, especially distal sites as the humerus [8]. Katyal et al. [17] reported a case series of 148 patients with HCC, where isolated bone metastasis as the initial manifestation was only seen in 9.5%. Two cases out of 148 patients have lytic lesions of the humerus. In literature, few cases have been published reporting solitary metastasis to the humerus as the first presentation of unknown primary HCC [9–13].

HCC spreads to the bone, mainly via the hematogenous route [18]. Some authors postulated that skeletal metastasis occurs via portal vein-vertebral vein plexuses, thus explaining the more common axial skeletal metastases [8]. However, distant, solitary metastasis does not support this explanation, as in our case to the humeral shaft. HCC is often hypervascular; however, if located in the bone, it can also be osteolytic. Therefore, hypervascularity should be taken into account before biopsy excisions since the procedure can cause an uncontrolled life-threatening hemorrhage, as reported by Hansch et al. [19] and Chen et al. [20] in humeral and a sternal metastasis of HCC, respectively. Although HCC should be included in the differential diagnosis of hypervascular and osteolytic lesions, bone metastasis from pheochromocytoma, renal cell carcinoma, thyroid gland cancer, and parotid gland cancer may show similar imaging findings [21]. Metastasis from HCC was considered rare in the past, and a result of cancer has an aggressive clinical course [22]. With the help of emerging therapeutic and palliative medicines, the outcome for patients with HCC has improved, and metastases are turning out to be progressively important considerations [23]. Extrahepatic metastases are more common in patients with advanced-stage primary tumors (>5 cm and large vessel vascular invasion), and its extrahepatic recurrence is uncommon after locoregional therapy (5 to 24%) [6]. The prognosis of HCC patients with extrahepatic metastasis is generally poor [24]. The median survival after the diagnosis of HCC is roughly 6 to 20 months [22]. Large tumor size, vascular invasion, poor functional status, and nodal metastases are all associated with a poor outcome [25, 26]. In the presence of bone metastases, the mean survival rate is severely reduced [27].

The extrahepatic metastasis of HCC was once regarded as a terminal event [25], and coexisting intrahepatic lesions usually are not treated by locoregional therapies like surgical resection or medical ablation [28]. In two large, randomized controlled trials, it was demonstrated that the multikinase inhibitor (MKI) sorafenib significantly prolonged survival in patients with advanced HCC, even when extrahepatic metastases accompanied the primary lesion. The Sorafenib Hepatocellular Carcinoma Assessment Randomized Protocol (SHARP) trial comparing sorafenib's effectiveness to placebo found that it prolonged mean survival by three months compared to placebo in patients with advanced HCC [29]. In the Asia-Pacific (AP) trial, the median overall survival was 6.5 months in patients treated with sorafenib, compared with 4.2 months in those who received a placebo [30]. Therefore, sorafenib was the first systemic therapy widely regarded as the standard treatment for patients with advanced HCC. Later, lenvatinib (also MKI) was approved as an alternative first-line therapy as it was confirmed to be noninferior to sorafenib in the REFLECT study [31]. Both these MKIs have an overall survival of less than 3 months relative to placebo with a considerable poor side effect profile. Ramucirumab, the vascular endothelial growth factor (VEGF) receptor inhibitor, and regorafenib and cabozantinib, the multitarget tyrosine kinase inhibitors (targets both MK and VEGF), have all been approved by the Food and Drug Administration (FDA) as single-agent second-line systemic therapy for patients who have failed sorafenib [32–35]. Due to the side effect profiles of the above choices, it warranted the need for further trials of therapeutic classes with similar or better survival benefit and acceptable side effect profile.

Currently, there are two classes of immune check inhibitors (ICIs) that are being investigated and clinically used as options in previously treated HCC patients with adequate performance status. These ICIs belong to the programmed death-ligand 1/programmed cell death protein 1 (PD-L1/PD-1) and cytotoxic T-lymphocyte-associated protein 4 (CTLA-4) inhibition pathways [36, 37] and have emerged as alternatives for patients with adequate performance status who progress on first-line therapy. The CHECKMATE 040 trial (PD-L1/PD-1) was aimed at assessing nivolumab's safety and efficacy in patients with advanced hepatocellular carcinoma with or without chronic viral hepatitis [38]. On September 22, 2017, the FDA approved nivolumab as an adjunct treatment for patients who have failed treatment with sorafenib. The KEYNOTE-224 trial (PD-L1/PD-1) was aimed at assessing pembrolizumab's efficacy and safety in patients with advanced hepatocellular carcinoma previously treated with sorafenib [39], and the FDA approved it in November 2018. These two PD-1 inhibitors have shown some important role in the management of advanced HCC as an adjunct treatment for patients who have failed treatment with sorafenib.

Drug combination treatments have found some success in HCC as a combination of ipilimumab (CTLA-4), and nivolumab (PD-L1/PD-1) was approved by the FDA in March 2020 [40]. The most recent exciting results published of the IMbrave150, a global, multicenter, open-label, phase 3 randomized trial of atezolizumab (PD-L1) plus bevacizumab (VEGF) in patients with untreated unresectable hepatocellular carcinoma showed that the combination was superior in prolonged OS and progression-free survival (PFS) as compared to sorafenib [41]. On May 29, 2020, the FDA approved this combination [42]. Considering all these advances, there is still a cohort of patients with advanced HCC, with which we face difficulties in evaluating the therapies they would gain from, owing to challenges in recognizing cellular and molecular markers [43].

## 4. Conclusion

SMUP is a rare, unusual metastatic tumor entity that imposes a diagnostic and therapeutic challenge. Incorporating genetic and molecular features in a multistage diagnostic workup is essential for characterizing the biological SMUP profile to direct therapeutic decisions. In this case, the unusual presentation raises the importance of a thorough preoperative evaluation for pathological fractures in otherwise asymptomatic patients. Patients with undiagnosed HCC may rarely have initial symptoms exclusively related to extrahepatic metastases. Although skeletal metastasis is not common with HCC but can involve the axial skeleton, rarely, the first manifestation of advanced HCC can be a solitary distant site metastasis in the appendicular skeleton. The patient can present as bone pain or pathological fracture, as in our case report. The prognosis of HCC patients with extrahepatic metastasis is generally poor, and in the presence of bone metastases, the mean survival rate is severely reduced. The MKIs sorafenib and lenvatinib, widely used as first-line systemic therapy, have an overall survival of less than 3 months relative to placebo with a considerable poor side effect profile. Recently, the development of ICIs has changed the therapeutic paradigm for advanced HCC. The most recent and exciting development is the FDA approval of combination therapy with atezolizumab plus bevacizumab in patients with untreated unresectable hepatocellular carcinoma. There is still a significant cohort of HCC patients who do not respond to ICIs despite the progress of ICIs, and the difficulty continues to identify cellular and molecular markers that might determine which patients will gain from these therapies. The authors of this case report do acknowledge that any molecular characterization from the specimen if available would have been helpful, potentially worth to gain insights for tailored target or personalized therapy for our patient.

## Data Availability

No data were used to support this study.
